# Autonomic Signature-Driven Anesthesia Depth Monitoring with Biomimetic Wearable ECG and Knowledge Graph-Augmented Deep Networks

**DOI:** 10.3390/s26113498

**Published:** 2026-06-02

**Authors:** Aoran Bao, Cheng Ding

**Affiliations:** 1 College of Smart Agriculture (College of Artificial Intelligence), Nanjing Agricultural University, Nanjing 210095, China; baoar@stu.njau.edu.cn; 2 College of Artificial Intelligence, Nanjing University of Aeronautics and Astronautics, Nanjing 211106, China

**Keywords:** graph neural network (GNN), depth of anesthesia, electrocardiogram (ECG), graph convolution layers

## Abstract

Considerable efforts have been devoted to accurately monitoring the depth of anesthesia to ensure patient safety during surgery. Traditional approaches typically rely on electroencephalogram (EEG)-based indices, such as the Bispectral Index (BIS), which require specialized equipment. In contrast, electrocardiogram (ECG) signals are widely available in clinical settings and can be conveniently acquired via wearable devices, while also exhibiting strong responsiveness to anesthetic agents. Inspired by biomimetic physiological regulation mechanisms, this study proposes a wearable-compatible ECG-based framework for depth-of-anesthesia detection that leverages autonomic nervous system characteristics and a knowledge graph-enhanced graph convolutional network (GCN). ECG recordings from 110 patients were preprocessed, and 20 anesthesia-related features were extracted, spanning morphological, statistical, spectral, heart rate variability (HRV), and entropy-based descriptors; feature selection methods identified 13 discriminative features. A patient-level knowledge graph was first constructed using the 88 training patients (1760 nodes), and test patient nodes were incorporated only after training was complete for inductive inference. Experimental results demonstrate that the proposed deep knowledge GCN achieves a test accuracy of 98.18% in distinguishing between awake and deep sleep anesthesia states, indicating that biomimetic, wearable-compatible ECG analysis combined with knowledge graph learning holds strong potential as a cost-effective alternative to traditional EEG-based anesthesia monitoring systems.

## 1. Introduction

Accurately controlling the depth of anesthesia remains one of the most challenging tasks in surgical practice. Approximately 300 million surgical procedures are performed worldwide each year, the majority of which require anesthesia [1]. Maintaining an appropriate anesthetic level is critical: insufficient anesthesia may lead to intraoperative awareness, whereas excessive anesthesia increases the risk of hemodynamic instability and delayed recovery. Clinical studies indicate that 20–30% of patients experience some degree of anesthesia mismanagement, characterized by either overly deep or insufficient anesthesia [2,3]. Although rare, intraoperative awareness occurs in approximately 0.1–0.2% of general anesthesia cases and is often associated with post-traumatic stress disorder (PTSD) [4]. Conversely, excessive anesthetic dosing has been reported to increase postoperative risks by approximately 23% (*p* < 0.05), including prolonged intubation, delayed recovery of consciousness, and increased mortality [5]. These findings highlight the necessity of precise anesthesia management to ensure patient safety and improve healthcare quality and resource utilization.

The Bispectral Index (BIS), derived from electroencephalogram (EEG) signals, is currently one of the most widely used clinical tools for monitoring anesthesia depth [6]. However, BIS monitoring has several limitations: it requires specialized equipment; is susceptible to artifacts from muscle activity, electrical noise, and surgical instruments; and may exhibit variability across anesthetic agents and patient populations [7,8]. Furthermore, its relatively high cost limits its applicability in resource-constrained settings. These limitations underscore the need for alternative monitoring approaches that are both reliable and accessible.

Electrocardiogram (ECG) signals, which reflect the electrical activity of the heart, offer a promising alternative. ECG monitoring is routinely performed in most operating rooms and can be continuously acquired without additional equipment. Previous studies have shown that general anesthesia modulates autonomic nervous system activity, manifested as changes in heart rate variability (HRV), QRS morphology, and non-linear ECG dynamics [9,10,11]. These physiological alterations can serve as reliable indicators of anesthesia depth, making ECG a practical and cost-effective modality for anesthesia monitoring, particularly in resource-limited environments.

In this study, we propose a framework for classifying ECG signals collected during general anesthesia into two clinically meaningful states—awake and deep sleep—based on corresponding BIS values. To ensure data quality, a multi-stage preprocessing pipeline is employed, including high-pass filtering for baseline drift removal, notch filtering (50/60 Hz) for power-line interference suppression, bandpass filtering (0.5–45 Hz), and wavelet-based denoising. This pipeline effectively removes noise and artifacts while preserving clinically relevant signal morphology, particularly the QRS complex, thereby improving the reliability of subsequent feature extraction and classification.

A comprehensive set of features is extracted from the preprocessed ECG signals to characterize patients’ physiological states under anesthesia. Time-domain features include the mean and standard deviation of RR intervals, root mean square of successive differences (RMSSD), and beat-to-beat variability metrics (NN50 and pNN50). Frequency-domain features, such as very-low-frequency (VLF), low-frequency (LF), and high-frequency (HF) power, along with the LF/HF ratio, reflect autonomic balance. Morphological features capture the mean and standard deviation of QRS amplitude and overall signal amplitude, indicating the stability of cardiac electrical conduction. Statistical features, including skewness, kurtosis, median, and interquartile range, describe signal distribution properties. Additionally, non-linear dynamic measures, such as Poincaré plot descriptors and sample entropy, quantify the complexity of cardiac dynamics. In total, more than 20 features are extracted from each signal segment to provide a comprehensive representation of physiological states.

By mapping these ECG-derived features to BIS-defined anesthesia states, our method demonstrates that ECG can serve as a viable alternative to EEG-based monitoring. Compared with conventional BIS devices, the proposed approach reduces cost and improves robustness in practical surgical environments with significant noise and interference. Overall, this framework offers a scalable and clinically applicable solution for anesthesia depth assessment.

A key limitation of existing ECG-based depth-of-anesthesia (DoA) methods is that they treat each patient’s physiological features as independent vectors, thereby ignoring inter-patient relationships. However, patients at similar anesthetic depths often exhibit consistent and quantifiable patterns in HRV suppression, QRS morphology, and spectral characteristics—patterns that become evident only when relational structures are considered.

To address this limitation, we propose an autonomic-signature-driven knowledge graph framework that encodes pairwise patient similarities based on ECG-derived biomarkers. A deep knowledge graph convolutional network (GCN) is employed to propagate and aggregate relational information across physiologically similar patients. This graph-based inductive bias represents a fundamental departure from conventional approaches. Experimental results demonstrate that the proposed framework achieves a classification accuracy of 98.18%, outperforming a 1D CNN baseline by nearly 19 percentage points, thereby confirming the importance of modeling inter-patient relational structures for anesthesia depth estimation.

## 2. Literature Review

### 2.1. EEG-Based Depth of Anesthesia Monitoring

EEG-based approaches have dominated DoA research owing to the direct relationship between cortical electrical activity and anesthetic states. Ronglin Li et al. [12] proposed a hybrid EEG feature set combined with a sparse denoising autoencoder (SDAE) and LSTM, outperforming traditional index methods such as alpha ratio and permutation entropy. Muhammad Ibrahim Dutt et al. [13] introduced a fractal feature-based MLP classifier using Stationary Wavelet Transform (SWT), achieving 96.8% accuracy with a reduced feature set. Qihang Wang et al. [14] developed Anes-MetaNet, a meta-learning deep neural network for classifying brain states under anesthesia, demonstrating superior performance over prior EEG-based methods on office-based anesthetic datasets. Meng Shi et al. [15] proposed a deep residual shrinkage network for DoA estimation from EEG, yielding a Spearman rank correlation coefficient of 0.9344. Yue Gu et al. [16] combined multiple EEG features with artificial neural networks (ANNs), reporting recognition accuracies of 84.4% for general anesthesia and 86.4% for wakefulness, with a BIS correlation coefficient of 0.892. Wala Saadeh et al. [17] developed an FPGA-implemented DoA processor using fast Fourier transform-based features (FBSE, SEF, beta ratio), maintaining 92.2% mean accuracy with only 1 s latency at 140 nJ per classification.

Despite their strong performance, EEG-based methods share fundamental practical limitations: they require dedicated electrode placement on the scalp, specialized acquisition equipment, and are sensitive to electromyographic artifacts and patient movement—factors that substantially restrict their applicability in resource-limited clinical environments and wearable scenarios.

### 2.2. ECG- and Multi-Modal Signal-Based Approaches

To overcome the equipment constraints of EEG-based systems, several studies have explored ECG and multi-modal physiological signals as alternative DoA indicators. Meghna Roy Chowdhury et al. [18] proposed a 10-layer CNN, accepting ECG and PPG heatmaps as inputs, and achieved 86% accuracy on data from 50 surgical patients, demonstrating that wearable-compatible signals can support high-precision DoA prediction at low costs. Sara Afshar et al. [19] introduced a combinatorial architecture fusing a bidirectional LSTM, attention layers, and an inception-module CNN, which achieved a mean absolute error of 4.3 ± 0.87 in BIS estimation with a 15% improvement in AUC over leading methods. Nooshin Bahador et al. [20] investigated fused EEG–ECG recordings for anesthesia state transition detection using ridge fusion time–frequency representations, reporting 94.14% precision with a 0.28 s forecast latency—demonstrating that multi-modal fusion can accelerate state transition detection while improving classification robustness. Oscar Mosquera Dussan et al. [21] applied entropy-based and complexity indices derived from biological signals to classify anesthesia depth, reporting high Pearson correlation between their entropy module indices and the Complexity Brainwave Index.

While these approaches reduce equipment dependency and expand the range of usable signal modalities, they share a critical architectural limitation: each patient’s physiological features are treated as an independent, isolated vector fed directly into a flat classifier. This paradigm inherently discards the inter-patient relational structure of autonomic nervous system responses—structured co-variations in HRV, QRS morphology, and spectral power—that are well-established signatures of anesthetic depth and only become accessible when patients are modeled in relation to one another.

### 2.3. Graph- and Deep Learning-Based Classification

Graph neural networks (GNNs) have emerged as a powerful paradigm for learning from relational data, and have recently been applied to biomedical signal analysis. By representing entities as nodes and their relationships as edges, GNNs enable information propagation through structured neighborhoods—a capability fundamentally absent in conventional sequence models. However, their application to ECG-based DoA monitoring remains largely unexplored. Existing graph-based works in adjacent domains [22,23,24] have demonstrated that encoding pairwise similarity among physiological samples into graph topology substantially improves classification performance over flat feature-vector baselines, particularly when inter-sample relationships carry discriminative information.

Motivated by this observation, the present work is—to our knowledge—the first to apply patient-level knowledge graph construction to ECG-based anesthesia depth monitoring. Rather than treating each patient’s ECG biomarkers as an independent data point, we construct a weighted knowledge graph in which nodes represent patient feature instances and edges encode pairwise physiological similarity via a Gaussian kernel. A deep knowledge GCN with residual connections and attention mechanisms then learns higher-order inter-patient interaction patterns from this graph, capturing the relational structure of autonomic responses that all prior ECG-based DoA methods have overlooked. This problem-driven design distinguishes our framework from existing approaches not merely in implementation, but in its fundamental modeling assumptions.

## 3. Methodology

**Figure 1:**
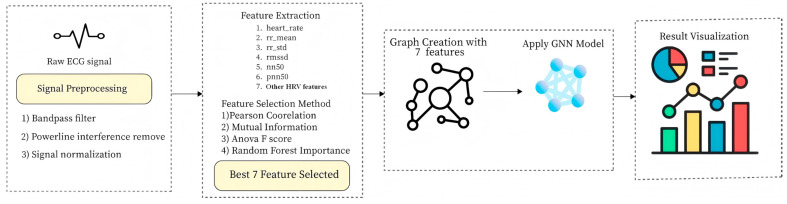
Proposed framework for anesthesia depth detection from ECG signals, illustrating the sequential pipeline of signal preprocessing, feature extraction, feature selection, and classification using a graph neural network (GNN).

An overview of the complete pipeline of this study is presented in Figure 1.

The proposed methodology for ECG-based anesthesia depth detection comprises five main components: signal preprocessing, feature extraction, feature engineering, knowledge graph construction, and graph-based classification. First, a preprocessing pipeline is applied to raw ECG signals from 110 patients, including baseline drift removal, power-line interference suppression, bandpass filtering, and wavelet denoising, to enhance signal quality and reduce noise.

Following preprocessing, representative ECG segments are selected, and a comprehensive set of physiologically meaningful features is extracted. These include amplitude-related metrics, energy- and power-based features, zero-crossing rates, statistical moments, spectral entropies, dominant frequency components, low-frequency/high-frequency (LF/HF) power ratios, heart rate variability (HRV) indices, RR interval statistics, and sample entropies. Each feature set is labeled as either awake or deep sleep based on the corresponding Bispectral Index (BIS) values.

In the feature engineering stage, three selection criteria—absolute Spearman correlation, mutual information, and ANOVA F-test—are employed to identify the 13 most discriminative features for anesthesia state representation. Based on these features, a large-scale knowledge graph is constructed, where each node represents a feature instance and edges encode similarity relationships, resulting in a graph with 2200 nodes and over 233,000 edges. This graph structure effectively captures complex relational patterns that are difficult to model using traditional machine learning approaches.

Finally, three graph learning architectures are evaluated on the constructed knowledge graph: a baseline knowledge graph convolutional network (GCN), an attention-enhanced GCN, and a deep knowledge GCN with residual connections. Owing to its ability to capture higher-order feature interactions, the deep knowledge GCN is adopted as the primary model. Furthermore, layer-wise node embedding analysis is conducted to enhance interpretability and to better understand the contribution of features to anesthesia depth classification.

### 3.1. Dataset Description

The dataset [25] utilized in this study comprises intraoperative physiological data collected from 110 patients undergoing general anesthesia at the National Taiwan University Hospital (NTUH). ECG signals were acquired using chest-mounted sensors connected to an MP60 anesthesia monitoring system (IntelliVue MP60, Philips Medizin Systeme Boeblingen GmbH, Boeblingen, Germany). The data were routed to a recording computer equipped with custom real-time acquisition software developed in Borland C++ Builder 6. The ECG signals were continuously sampled at a high resolution of 500 Hz. Simultaneously, EEG and PPG data were continuously recorded at 128 Hz. Furthermore, vital clinical parameters including the Bispectral Index (BIS)—serving as the primary reference for sedation depth—along with heart rate (HR), pulse rate (PR), blood pressure (BP), and blood oxygen saturation (SpO_2_) were recorded at 5 s intervals. All raw data were exported in .mat format for offline signal processing and analysis using MATLAB R2023b (MathWorks, Natick, MA, USA).

**Figure 2:**
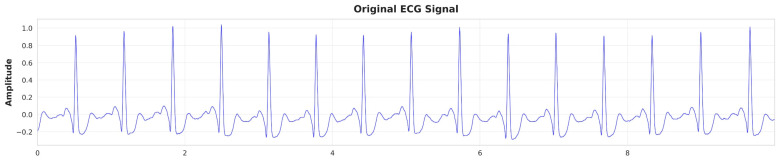
Overlay of ECG signal.

To establish reliable ground truthlabels for model training, reference curves reflecting the continuous assessment of anesthesia depth were generated based on the consensus of five experienced clinicians (as illustrated by the representative ECG overlay in Figure 2). To ensure rigorous evaluation and prevent data leakage, the dataset was partitioned at the patient level. Specifically, all ECG segments belonging to a given patient were assigned exclusively to either the training set or the test set, with no overlap. The 110 patients were split into a training cohort (88 patients, 80%) and a strictly held-out test cohort (22 patients, 20%). This patient-level splitting strategy explicitly prevents the model from observing data from the same patient during both training and testing, ensuring that the reported classification performance accurately reflects the model’s ability to generalize to entirely unseen patients in a realistic clinical setting. Specifically, the knowledge graph was constructed exclusively from training patient features; test patient nodes were introduced to the graph only after model training was finalized, with no weight updates performed during inference, thereby ensuring a fully inductive evaluation protocol.

### 3.2. Signal Preprocessing

**Figure 3:**
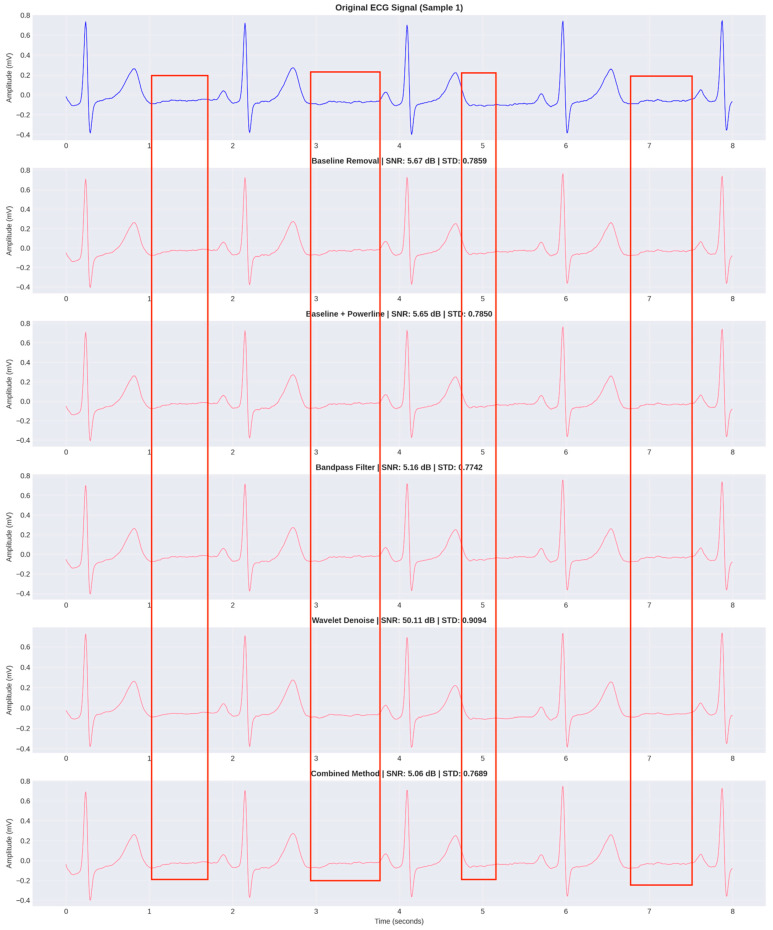
Signal preprocessing pipeline applied to a representative ECG segment, illustrating the sequential effects of baseline removal, baseline + power-line filtering, bandpass filtering, wavelet denoising, and the combined method. Red boxed regions highlight areas where inter-stage differences are most discernible.

Before extracting features, the raw ECG signals were preprocessed to eliminate noise, artifacts, etc., thus making the data more consistent. The preprocessing pipeline is as follows: baseline removal, baseline + power line, bandpass filter, and wavelet denoising. The visualization of the raw signal and the processed signal is shown in Figure 3.

ECG preprocessing is essential for improving signal quality by reducing baseline drift, suppressing power-line interference, attenuating high-frequency noise, and preserving clinically relevant ECG morphology [26]. In this study, four preprocessing operations were applied in sequence: baseline wandering removal, power-line interference suppression, bandpass filtering, and wavelet-based denoising. Preprocessing usually aims to obtain an accurate ECG signal that has retained the QRS waveform, decreased distortion as much as possible, increased SNR, and improved the reliability of subsequent feature extraction.

Baseline wandering is removed by means of a high-pass filter operation [27]. By applying a high-pass filter with an appropriate cut-off frequency, this transforms the raw ECG signal x(t) into its baseline-corrected form $x_{bw}(t)$. The filter’s transfer function is written as (1) [28]. This operation eliminates the slow fluctuation of respiratory rate and electrode movement; therefore, the amplitude-related features after are still meaningful for physiological analysis.

(1) $$x_{bw}(t)=x(t)-(x(t)*h_{lp}(t))$$

where * represents the convolution and $h_{lp}(t)$ is a low-pass smoothing kernel for estimating the drifting baseline.

A notch filter was employed to eliminate 50/60 Hz power-line interface [29], whose frequency response is shown in (2). This type of filter can selectivity reduce a narrow band sinusoidal interference caused by the electrical mains without affecting other parts of the ECG signal, such as QRS waves [30].

(2) $$H_{notch}(z)=\frac{1-2cos(2\pi f_{0}/f_{s})z^{-1}+z^{-2}}{1-2rcos(2\pi f_{0}/f_{s})z^{-1}+r^{2}z^{-2}}$$

where $f_{0}$ is the interference frequency, $f_{s}$ is the sampling frequency, and r controls notch sharpness.

A bandpass filter [31] is used to maintain the primary ECG spectral components at approximately 0.5–45 Hz, and a band-limited signal is obtained. This removes electromyographic noise, motion artifacts, and the residual baseline component. Bandpass filtering is widely recommended for clinical QRS detection and morphological analysis [31].

(3) $$x_{bp}(t)=x(t)*h_{bp}(t)$$

where $h_{bp}(t)$ denotes the impulse response of the bandpass filter.

To further enhance the denoising performance, based on Equation (4) of the wavelet threshold function, the filtered signal is decomposed into several scale coefficient groups. This eliminates stochastic and high-frequency noise components while preserving sharp discontinuous information, such as QRS peaks.

(4) $$W_{x}(a,b)=\int_{-\infty }^{\infty }x(t)\psi_{a,b}(t)dt$$

where $\psi_{a,b}(t)$ is the scaled and shifted mother wavelet.

Thresholding is then performed according to the classical wavelet shrinkage rule in (5) to eliminate noise-dominant coefficients and retain high-amplitude coefficients that represent the ECG structure.

(5) $$\hat{W}(a,b)=\left\{\begin{array}{cc} 0, & |W_{x}(a,b)|<\lambda \\ W_{x}(a,b)-\lambda \cdot \mathrm{sign}(W_{x}(a,b)), & |W_{x}(a,b)|\geq \lambda \end{array}\right.$$

Inverse wavelet reconstruction technology is used to obtain a final denoised ECG signal [32]. Compared with the combined preprocessing strategies, the SNR is improved, amplitude characteristics are stable, and it can better reflect the physiological changes caused by anesthesia.

### 3.3. Feature Extraction

After the preprocessing of the electrocardiogram signals, a set comprising 20 features is extracted to describe the changes in physiology related to the degree of general anesthesia. These features are morphological, statistical, spectral, and heart rate variability (HRV), which can reflect the cardiovascular autonomic response to anesthesia. Morphological features are the wave shape and amplitude changes; statistical and complexity features describe the distribution of signals and their disorderliness; spectral features reflect frequency; and HRV indices show sympathetic nerve alterations. Together, these characteristics can produce an abundant expression to identify the awake and deep sleep states. Table 1 shows all these features, along with their respective formulas and explanations.

Together, these characteristics offer an all-round description of ECG signals in the time–frequency domain, reflect changes in signal amplitude and complexity, and retain spectrum information related to the autonomic nervous system reflecting the depth of anesthesia.

### 3.4. Feature Selection

A total of 20 ECG features were extracted, and then feature selection was performed to determine which features were most discriminative and non-redundant for classifying anesthesia depth. Dimensions were decreased to improve model interpretability and reduce the probability of overfitting. The three selection methods used in this paper are: absolute Spearman correlation, mutual information score, and ANOVA F-score.

Absolute Spearman correlation calculates the degree of monotonic relationship between each feature and the target anesthesia state by computing the rank-based correlation coefficient. Features with larger absolute correlation values are considered more related because they have a greater connection to the awake or deep sleep state. Spearman’s rank correlation coefficient ρ is given by:

(6) $$\rho =1-\frac{6\sum_{i=1}^{N}d_{i}^{2}}{N(N^{2}-1)}$$

where $d_{i}$ is the difference between ranks of the i-th feature value and its corresponding target label, and N is the number of samples.

The mutual information score quantifies the dependence of a feature on the target label as an increase in our knowledge about the anesthesia state after learning that feature. Features with higher mutual information are more helpful for class separability. Mathematically, mutual information I(X;Y) is given by:

(7) $$I(X;Y)=\sum_{x\in X}\sum_{y\in Y}p(x,y)log\frac{p(x,y)}{p(x)p(y)}$$

where p(x,y) is the joint probability distribution of feature X and target Y, and p(x) and p(y) are their marginal distributions.

ANOVA F-score is the ratio of variance between classes and variance within classes for each feature. Features with a higher F-score are more likely to discriminate between awake and deep sleep states. The F-score is calculated as follows:

(8) $$F=\frac{\sum_{c}n_{c}{\left({\bar{x}}_{c}-\bar{x}\right)}^{2}/(C-1)}{\sum_{c}\sum_{i\in c}{\left(x_{i}-{\bar{x}}_{c}\right)}^{2}/(N-C)}$$

where $n_{c}$ is the number of samples in class c, $\bar{x}$ is the class mean, $\bar{x}$ is the overall mean, C is the number of classes, and N is the total number of samples.

**Figure 4:**
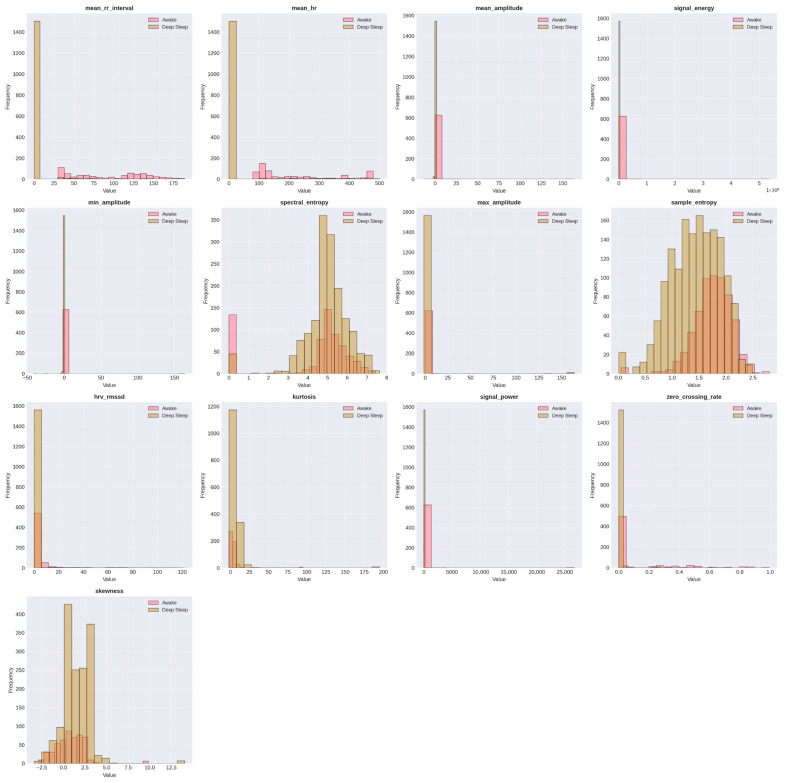
Correlation between the top 13 selected ECG features and anesthesia state (awake versus deep sleep). Features were ranked by Spearman correlation, mutual information score, and ANOVA F-score in combination. Positive correlations indicate features that increase as anesthesia depth increases; negative correlations are the opposite. This visualization presents the most discriminative features utilized in knowledge graph construction.

Based on the combined rank of these three approaches, the top 13 features were selected. The selected feature set is shown in Figure 4.

Among them, the selection of these specific characteristics is an optimized balance that retains the essential distinctions for anesthesia state identification and removes redundant data. Therefore, this will help improve the construction efficiency of the ECG knowledge graph and provide structural support for enhancing the prediction accuracy of the following graph-based learning framework.

### 3.5. Knowledge Graph

Building a patient-feature knowledge graph can help us formally examine the relational structure among patients’ basic information and physiological data under anesthesia. The following is a chart containing the top 15 discriminative ECG features to ensure that the model focuses only on the most related indicators of different anesthetic depths. The graph is defined as an undirected weighted graph.

**Figure 5:**
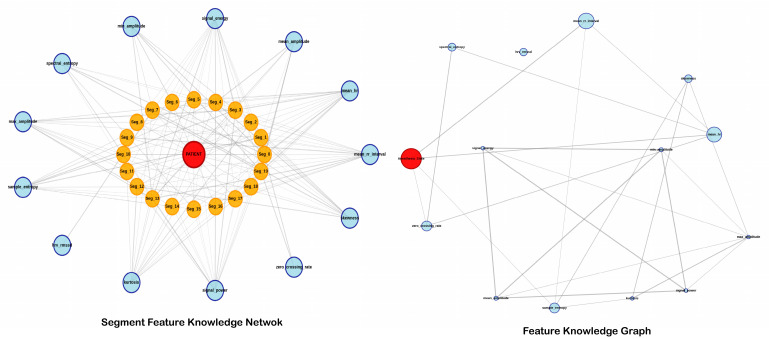
Example visualization of the constructed knowledge graph for a single patient, illustrating feature relationships and similarity-based connections to other patients.

(9) G=(V,E,W)

where V is a set of nodes, E represents the edges connecting these nodes, and W is an edge-weight matrix. The relational structure of the knowledge graph is shown in Figure 5.

There are two kinds of nodes: (1) patient nodes and (2) feature nodes. Each node of the patient is an ECG sample whose features have been extracted. Formally, each patient node $v_{i}$ is expressed as a feature vector:

(10) $$x_{i}=[f_{i1},f_{i2},\ldots ,f_{iK}]$$

where K = 15 is the number of selected features, and $f_{ij}$ represents the value of the j-th feature for the *i*-th patient.

Edges connecting patient nodes are generated according to the pairwise similarities of their feature vectors. For any two training patients, the similarity is calculated as i and j with a Gaussian kernel:

(11) $$S(i,j)=\exp\left(-\frac{∥x_{i}-x_{j}{∥}^{2}}{2\sigma^{2}}\right)$$

where σ controls the degree of similarity. Patients with a higher degree of similarity have more edges. When an edge is added:

(12) (i,j)∈E if S(i,j)≥τ

where τ is the similarity threshold used to prevent noisy or meaningless edges.

The weight of each edge encodes the similarity strength:

(13) $$W_{ij}=S(i,j)$$

resulting in a weighted adjacency matrix.

To stabilize GCN-based learning, the normalized adjacency matrix is calculated as follows:

(14) $$\tilde{A}=D^{-\frac{1}{2}}(A+I)D^{-\frac{1}{2}}$$

where I is the identity matrix and D is the degree matrix, which is defined by:

(15) $$D_{ii}=\sum_{j}(A_{ij}+I_{ij})$$

This type of graph structure can capture the relationship among patients caused by physiological patterns, thereby allowing learning of anesthesia-specific embeddings. Patients with similar ECG characteristics during general anesthesia are highly interconnected, and graph neural networks can propagate this significant clinical information among neighbors.

### 3.6. Proposed Deep GNN

We designed a GNN model to mine intricate patterns and relationships among nodes in the graph. The specific details of the proposed GNN model, such as how to represent inputs, pass messages, embed nodes, and construct graphs, are as follows.

#### 3.6.1. Input Representation

A graph input can be expressed as a tuple that consists of node features, edges, and edge weights [25]:

(16) G=(V,E),

where V is a set of nodes (vertices) and E is a set of edges. The node features matrix, $F\in {\mathbb{R}}^{d\times |V|}$, which captures the attributes of each node, has a number of feature dimensions as d and a quantity of nodes equal to n_nodes.

The message-passing process starts with an embedding for each node given by μ:

(17) $$F_{\mu }^{0}=x_{\mu },$$

where $x_{\mu }$ is the initial features of node μ. The edges and their corresponding weights show how the nodes are linked, as well as the strength of these links, thereby organizing the data in an orderly manner to support the subsequent graph [27].

#### 3.6.2. Message-Passing Layer

A message-passing layer serves as a primary part of graph neural networks. Node representation is updated through iterative updating of the neighbor nodes’ information. The above iteration captures the dependence and interaction of the network.

During iteration i, the embedding of a node μ is updated by combining its previous embedding with the aggregated messages from its neighbors [43].

(18) $$\begin{array}{ccc} F_{\mu }^{\left(i+1\right)} & = & {\mathrm{Update}}^{i}\left(F_{\mu }^{i},{\mathrm{AGGREGATE}}^{\left(i\right)}\left(\left\{X_{\nu }^{\left(i\right)}|\nu \in N(\mu )\right\}\right)\right)\\ & = & {\mathrm{Update}}^{i}(X_{\mu }^{i},m_{N(\mu )}^{\left(i\right)}) \end{array}$$

Here, ${\mathrm{AGGREGATE}}^{\left(i\right)}$ collects embeddings from the neighbors of node μ to form the message $m_{N(\mu )}^{\left(i\right)}$. The function Update^i then combines this message with the previous embedding $X_{\mu }^{i}$ to produce the new embedding $F_{\mu }^{\left(i+1\right)}$. This ensures that each node’s embedding captures both its own features and the structural information from its local neighborhood.

#### 3.6.3. Node Embedding and Graph-Level Formulation

After i iterations of message passing [44], the final node embeddings $Z_{\mu }=x_{\mu },\nabla_{\mu }\in V$ encapsulate the aggregated information from each node’s neighborhood. These embeddings can be applied to the following downstream applications; for example, node classification and link prediction. Pooling operations are performed on graphs for tasks such as graph classification to obtain their embeddings.

Then, the overall graph-level formulation of the model can be given as below:

(19) $$F^{i}=\sigma \left(AF^{\left(i-1\right)}W_{\mathrm{neighbour}}^{i}+F^{\left(i\right)}W_{\mathrm{self}}^{\left(i\right)}\right)$$

Here,

- A is the adjacency matrix representing the graph structure;
- $W_{\mathrm{neighbour}}^{i}$ and $W_{\mathrm{self}}^{\left(i\right)}$ are learnable weight matrices representing contributions from neighbors and the node itself, respectively;
- σ is the activation function.

**Figure 6:**
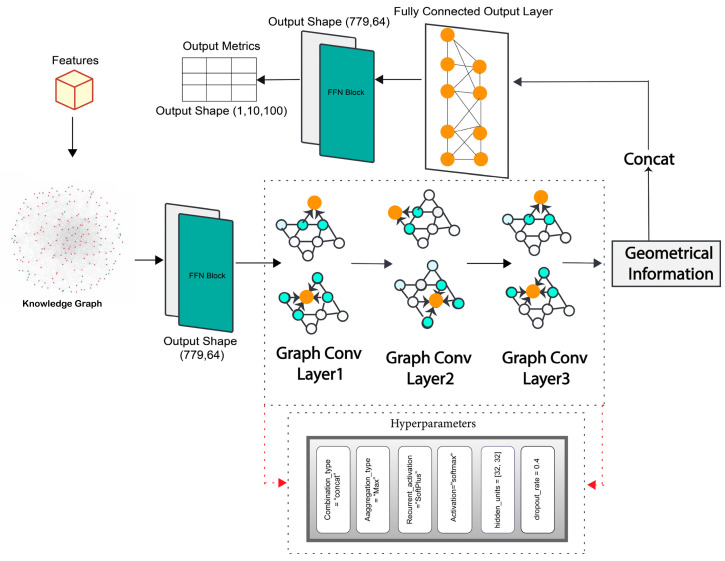
Proposed GNN architecture.

The formulated implementation of the GNN using sparse matrix operations is efficient and can be applied at a larger scale for graphs. Figure 6 presents the proposed structure of the GNN model.

The proposed GNN model fuses various parts in order to enhance the accuracy of its predictions. First, the characteristics of the nodes are input into several graph convolutional networks (GCNs). There are aggregation functions, a combination function, an activation function, recurrent activation, hidden units, and dropout in each GCN layer. The aggregation function obtains data from the nodes adjacent to it, and then the combination function merges this data with the node’s own state. Activation functions make things non-linear to help the model understand complex relationships. In the model we suggest, there are three GNN layers, and each one is meant to improve the node’s embeddings over time. The GCN layer sends these messages to the next GCN layer and updates node embeddings accordingly. Therefore, through the hierarchy, it can observe both local and global structures within the graph at once. The FFN block makes a final prediction after the GNN layers have combined the node embeddings. We will adjust the hyperparameters to make them better. The number of hidden units is set to [35], the learning rate is reduced to 0.01, and the batch size is increased to 64. We prevent overfitting by using a dropout rate = 0.3. We employ an SGD optimizer and an ELU activation function. A combination function of ConvLSTM1D is used to process time-series inputs, thereby making it easier for the model to identify complex dependencies.

## 4. Results Analysis

The effectiveness of the proposed GNN Model in classifying two anesthesia states (awake and deep sleep) systematically was evaluated in order to verify the high precision and stability of the model from multiple dimensions. In particular, an ablation study was conducted to determine which feature contributed more; subsequently, a confusion matrix was plotted to illustrate the class-wise prediction situation, and several quantities were calculated separately: accuracy, precision, recall, F1-score, and AUC. Loss and AUC curves were used to observe the changes in model convergence and discriminative ability during training to determine the effect at different classification thresholds, thereby finding an optimal decision surface. Finally, the performance of the proposed GNN model was compared with those of other baseline and state-of-the-art models to demonstrate its superiority in predicting post-anesthesia states.

### 4.1. ECG Preprocessing Evaluation

To ensure the reliability of downstream anesthesia state classification, an organized preprocessing pipeline was constructed for noise suppression, baseline drift correction, and enhancement of clinically relevant waveforms. Two quantitative metrics are used to assess the quality of processed signal noise ratio (SNR) and standard deviation, STD. SNR is a measure of the clarity and recoverability of the underlying cardiac waveforms after noise reduction; conversely, STD reflects the fluctuation state of the signal’s amplitude changes, indicating the stability condition of the corrected waveform. These complementary measures can be compared more subjectively among different preprocessing methods in terms of both noise reduction and signal smoothness.

**Figure 7:**
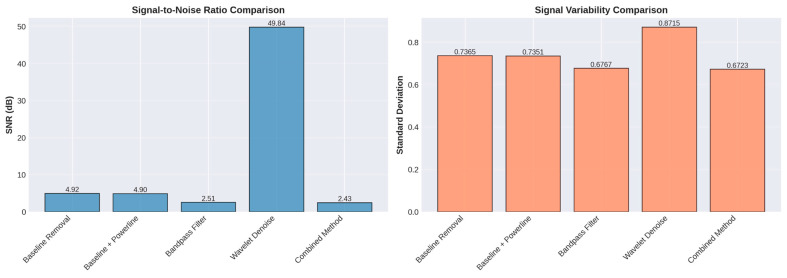
Quantitative comparison of ECG preprocessing methods using signal-to-noise ratio (SNR) and standard deviation (STD). Higher SNR and lower STD values indicate superior noise suppression and improved waveform stability.

As shown in Figure 7, different preprocessing techniques cause varying extents of damage to the completeness of an ECG signal. Here, a high-quality signal is defined as an increase in the SNR value and a decrease in the STD. There are specific indicators that must be reached; otherwise, these will not be strong enough to help improve the subsequent assessment accuracy of the anesthesia state.

### 4.2. Feature Selection Evaluation

**Figure 8:**
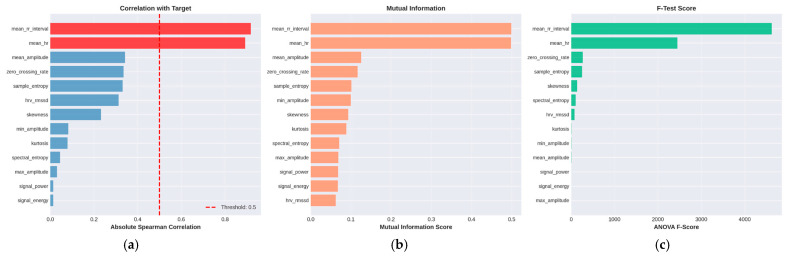
Feature selection evaluation using three metrics: (**a**) absolute Spearman correlation, (**b**) mutual information score, and (**c**) ANOVA F-test score. These analyses highlight the most physiologically relevant and discriminative ECG-derived features for differentiating anesthesia states.

We selected predictors that can better reflect the extent of anesthesia via multiple rounds of selection. To achieve a three-dimensional assessment for all combinations of features, the following were used: rank correlation, information mutual relationship, and statistic discrimination. The results of this all-round evaluation reflect that, at many different points, each examinee has been evaluated, as can be seen in Figure 8.

To verify whether the effectiveness of our feature selection was satisfactory from multiple perspectives, we have arranged these according to absolute Spearman correlation, as shown in Figure 8; that is, to show how much the selected features are related to changes in depth of anesthesia. From the data, it can be seen that mean_rr_interval and mean_hr show a very significant monotonic relationship with the anesthetic state. Mean_rr_interval, mean_hr, and mean_amplitude are more prominent among all these characteristics; when we try to determine how a person’s body changes under general anesthesia, these play a more crucial role. As shown in the middle part, these particular indicators have achieved the maximum mutual information scores. This confirms their great importance at catching those tricky, non-linear dependencies which simpler tests may miss. As predicted, the factors are, in fact, manifestations of changes in the autonomic nervous system and cardiovascular reactions at varying degrees of sedation. They merely perform the optimal task of encoding the complex physiological signatures that we require. On the right-hand side is the ANOVA F-test result, indicating how much each feature helps to distinguish between the awake and sleep states; among them, mean_rr_interval and mean_hr achieved the highest level of statistical significance, and their performance were verified by multiple selection criteria. We have organized a relatively complete feature set. By combining linear and non-linear physiological indicators, this model does not lack any patterns that should be present. And this two-tier analytical framework is precisely the driver of the high accuracy we observed in classifying anesthetic depth.

### 4.3. Ablation Study

A total of eight experiments were carried out to improve the performance of the proposed GNN model. All aspects of each test were taken in turn and the best configuration was selected. Table 2 shows the results of the eight instances of ablation research.

Table 2 presents the results of the hyperparameter ablation experiments conducted to optimize the proposed model. A range of hyperparameter categories were systematically examined, including the number of convolutional layers, hidden units, combination type, activation function, optimizer, learning rate, dropout rate, and batch size. For each category, one parameter was varied at a time while the remaining settings were held at their default values, and both test accuracy and average training time per optimization step were recorded. The best single configuration result of 91.46% test accuracy was observed at a batch size of 64, with a dropout rate of 0.3, ELU activation function, ConvLSTM1D combination, two hidden layers of 64 units each, SGD optimizer, and a learning rate of 0.01, demonstrating the sensitivity of model performance to individual hyperparameter choices.

It should be noted that the accuracy values in Table 2 reflect single variable ablation results and do not represent the performance of the fully assembled system. The final deep knowledge GCN reported in Table 3 integrates all identified optimal hyperparameters simultaneously alongside the complete knowledge graph structure. It is this joint optimization combined with the relational modeling of inter-patient physiological similarity that accounts for the performance improvement from 91.46% to 98.18%.

### 4.4. Performance Analysis of the Proposed GNN Model

**Figure 9:**
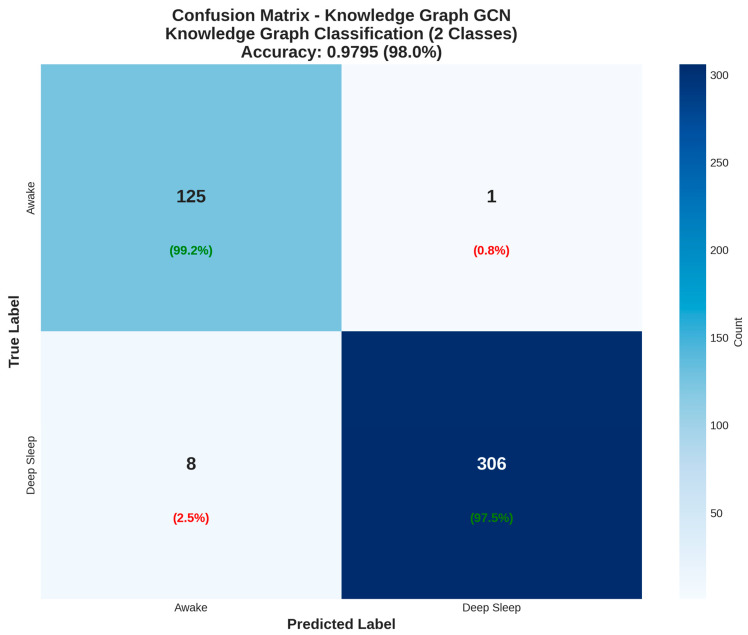
Confusion matrix of the proposed GNN model.

To ascertain whether the model possesses genuine category identification capabilities for each anesthesia stage, Figure 9 visualizes a detailed view showing which classes are predicted more precisely. And according to the chart, this framework shows overall performance in a refined selection of ECG-derived indicators, as well as having micro-scale reliability at all levels of sedation.

The knowledge graph GCN model has a higher degree of precision for the awake and deep sleep states. The reason why it has stronger discrimination ability is mainly because the graph’s rich structure can extract more useful information related to anesthesia degree from physiological characteristics compared with traditional methods.

By analyzing the confusion matrix, it was found that the error rate of this model is extremely low. Among all the data, only one awake sample was incorrectly classified as deep sleep, and there were only eight false alarms among the deep sleep samples. These were transformed into positive rates as 99.2 percent and 97.5 percent.

By correctly classifying 125 awake and 306 deep sleep instances with such a small error rate, it can be determined that the model was not overfitting the training data but had truly learned these two states. Considering that these indices have close connections among them, we therefore determined that adopting the graph-based model was an effective technical approach to address this.

**Figure 10:**
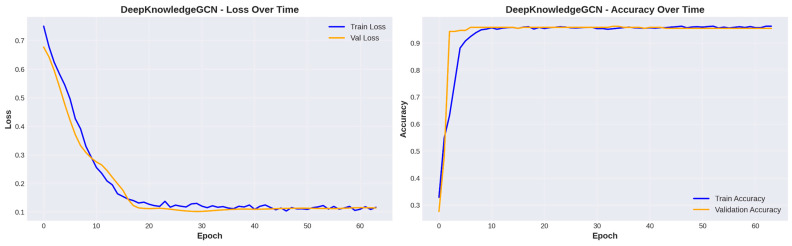
Loss and accuracy curves of the GNN model.

The trend in Figure 10 most accurately reflects the learning behavior of the model. Regarding the reason why the following curves are not only index numbers but also represent different times during training, we can observe a noticeable peak in the initial curve of accuracy and loss; therefore, it is likely that our model has rapidly identified the essential attributes associated with this dataset.

A concurrent increase in both training and validation set size has particular promise. It has been confirmed that the optimization process is stable, and more crucially, that the model is learning patterns which generalize well on unseen data. Even if there is still a small difference at the peak, it is very likely that the system has achieved its desired level of performance and will not drift into overfitting.

The training curve of the deep knowledge GCN shows a relatively stable learning effect during the optimization process. In the loss curve, both training and validation losses fall quickly in the early stages of training before fluctuating smoothly. Finally, they all tend to be close to 0.10. The two curves remaining close indicates that the model has good generalization capability and does not exhibit overfitting.

We can observe a similar pattern from the accuracy curve; it rises sharply during the initial several iterations and soon exceeds 0.90. After this, both remain stable and are close to the final performance peak. And these curves show that the model has learned well, converged normally, and maintained a stable relationship between the training set and the validation set.

### 4.5. Comparison with 1D CNN Models

To determine the extent to which our proposed GNN can classify anesthesia depth better than a typical 1D CNN. Competition is not only an index race among several models but also fails to thoroughly examine why there are such differences.

The table shows the comparison of the four different models, which are the 1D CNN, the knowledge graph GCN, the enhanced knowledge GCN, and the deep knowledge GCN, referred to as “Best” in this paper. The deep knowledge GCN has consistently shown better prediction results, and its accuracy, sensitivity, specificity, positive predictive value (PPV), negative predictive value (NPV), and F1-score have all remained close to 98 percent. The model can fully distinguish between different classes, and its performance is excellent. Using a knowledge graph GCN approach has yielded some expected outcomes; roughly 97 percent to 99 percent of the performance metrics are satisfied. The 1D CNN is at a moderate level of performance, while the enhanced knowledge GCN has the weakest result among the four; it shows relatively low accuracy and a high error rate, including a higher false positive rate (FPR), false discovery rate (FDR), and false negative rate (FNR). In summary, it can be shown that bringing in a more substantial knowledge graph structure at the model level has led to a marked improvement in predictive performance.

### 4.6. Model Learning Dynamics and Decision Boundary Analysis

**Figure 11:**
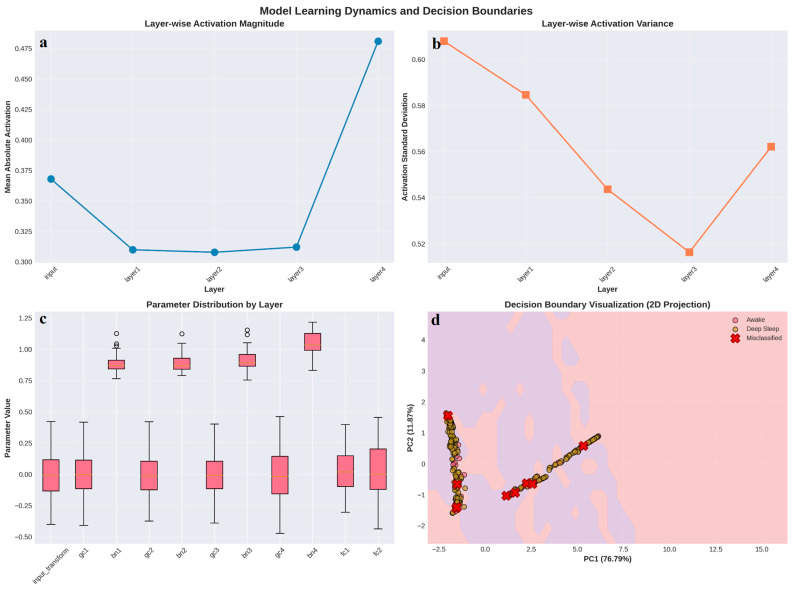
Model learning dynamics and decision boundaries: (**a**) layer-wise activation magnitude, (**b**) activation variance, (**c**) parameter distribution by layer, and (**d**) 2D decision boundary visualization using PCA. In panel (**d**), different colored regions indicate the decision regions assigned to different anesthesia stages by the proposed model, while the plotted points represent samples projected onto the first two principal components.

We decompose the internal mechanism of our GCN in Figure 11, and it shows that ECG features are transformed into different states of anesthesia. The most telling result is the 2D decision boundary projection; that is, it is feasible to separate the awake and deep sleep states clearly. To illustrate how the model achieves such a classification, Figure 5 monitors three internal characteristics of the activation function at different layers: magnitude (activation), variance (activation variance), and density of parameters. By observing this change, we can determine that the model is gradually enhancing its generalization ability during training and therefore simplifying how we determine what decision the GCN is making.

The mean absolute activation decreases from the input layer (0.367) to layer 2 (0.307), indicating that the features are being compressed at this point. Although there has been a small rise at layer 3 (0.313), it has risen sharply to 0.481 by layer 4. This terminal surge indicates that the deeper-layer features have more significant separability; therefore, it is in line with the model’s high classification accuracy (98.18%).

We can observe that there is a general stability in feature encoding as we move deeper in the network; the activation variance decreases from 0.609 at the input layer to a minimum of 0.515 by layer 3. The decline of this downward trend is expected to reduce internal noise. Surprisingly, this deviation bounces back slightly at layer 4 (0.557), and it is probably because the model increases the class-specific activation to be prepared for the final classification. From a weight perspective, the box plot shows that although the layers still remain centered with moderate spread, there are some blocks, specifically g4 and fc1, that extend to 1.2 for their positive outliers. These “heavier” weights are essential because they indicate which characteristics of the data the model depends more heavily on for class discrimination. The model can achieve a very clear distinction among the awake and deep sleep classes, as shown in the two separate clusters of the PCA 2D projection. This is primarily due to the first two principal components, where PC1 and PC2 account for 76.79 percent and 11.87 percent of the variance, respectively. We can see that the clusters are relatively clear, where only a small number of misclassified samples were found, marked in red as “X”, having into the boundary area, and this boundary is still relatively smooth, indicating that the model has learned generalized features rather than overfitted noise. The above high results are supported by the early-layer behavior, which is around 0.5–0.5, and has been in a stable, smooth state throughout the entire initial stage of the network.

### 4.7. Prediction Probability Analysis

**Figure 12:**
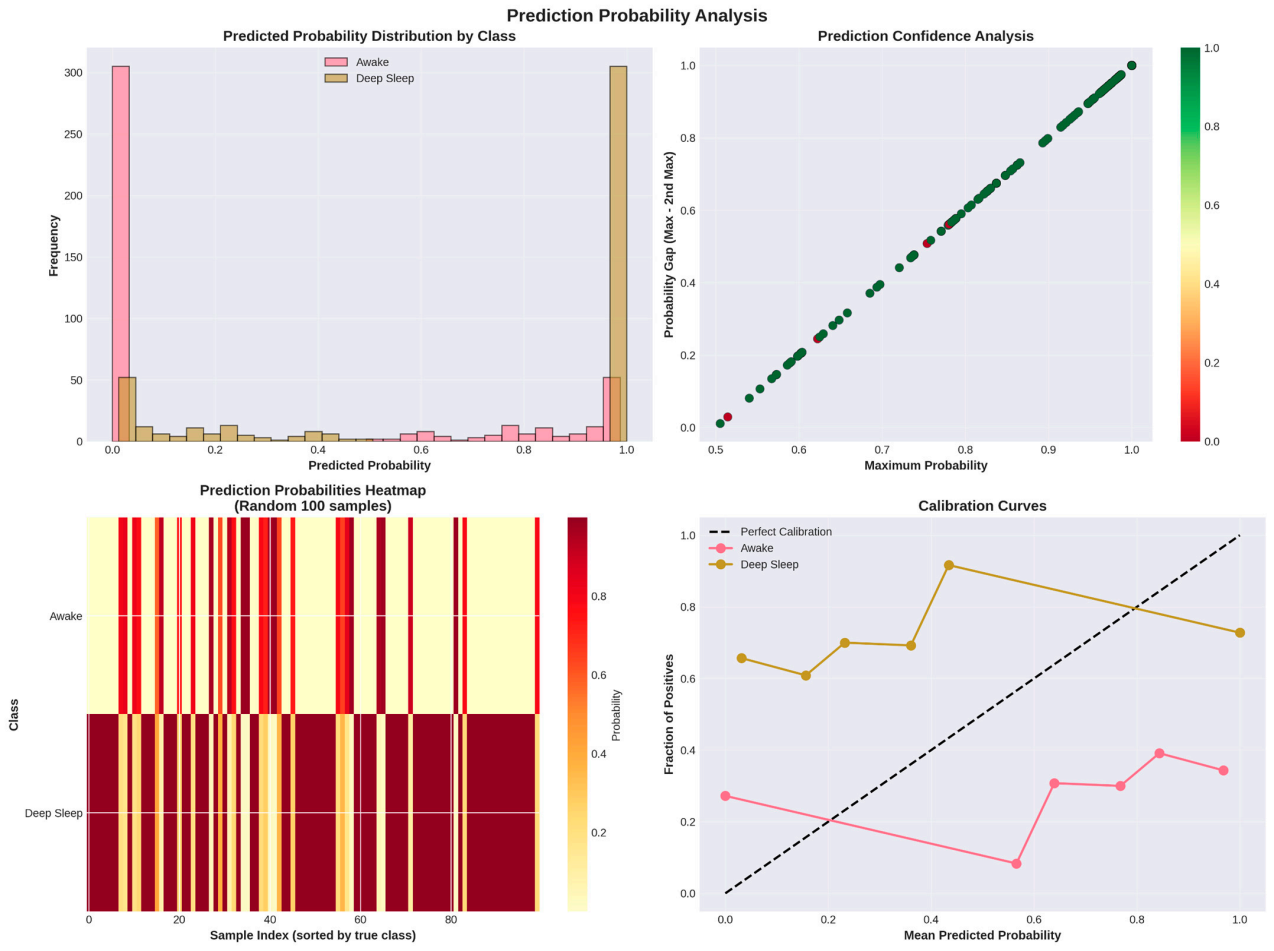
Prediction probability analysis. (**Top left**) Class-wise predicted probability histograms. (**Top right**) Prediction confidence and probability gap analysis. (**Bottom left**) Heatmap of prediction probabilities for 100 random samples. (**Bottom right**) Calibration curves comparing predicted vs. actual fraction of positives.

Figure 12 shows a summary of the prediction probability behavior of the model, including class probability distribution, confidence analysis, probability heat map and calibration curve. Not only does it show the label information, but it also reflects the “certainty” of each decision, that is, how the model determines the awake or sleep state, including the prediction confidence degree and whether predicted probability value has higher correspondence with the actual result probability.

The model’s ability to clearly distinguish among states is most evident by examining the probability histogram in the upper-left corner where predictions of deep sleep are overwhelmingly confident. There is a substantial peak exceeding 300 samples in the range from 0.95 to 1.0; therefore, the network rarely hedges its bets. The awake samples are also clustered closely around 0 and 0.1 on the other end of the scale. The “bipolar” distribution is thus confirmed to be very distinct, meaning that there will be little overlap among samples from the two classes.

The model’s decision is relatively inflexible. Looking at the prediction confidence analysis, there is nearly a perfect linear relationship between the maximum predicted probability and the “gap” to the runner-up class. This is not merely an occasional occurrence, but instead suggests that in most cases of the model selecting a winner, it does so overwhelmingly. That is to say, among them, the range from 0.85 to 1.0 is included, and the probability gap ranges between 0.6 and 1.0. There is a significant gap in most of these cases, as the network is not torn between two options. Only a small number of “borderline” samples are observed in the range of 0.5 to 0.6, at which point the model is indeed less certain.

A probability heatmap at the bottom left shows a class-wise separation for 100 randomly selected samples. The awake samples have warm colors almost entirely in the low end of the range from 0.0 to 0.2, while the deep sleep samples generally reach a high intensity at around 1.0. Only a small number of misclassified or low-confidence cases appear as stripes in contrast.

Finally, the calibration curve at the lower right shows overall information to help us determine how much trust we should place in these probability score values. For deep sleep, the model is close to the observed fraction and tracks the diagonal reference line very well, particularly in the range of 0.6 to 0.9. It is therefore reliable. In the awake class, we show a bit more deviation in the neural network, which is generally slightly underconfident between 0.2 and 0.6, giving a lower estimate of the probability of that state. However, these are minor differences. Overall, calibration remains relatively stable and aligns reasonably well with the general accuracy of the model at around 98.18%.

Through synthesis of these three perspectives via the following model—that is, threshold sensitivity, class-wise confusion, and across-subject variance—we transcend singular accuracy metrics for a more precise picture of the model’s conduct in practice. Model stability is reflected more prominently by strict confidence filtering; after that, if the upper limit of this threshold increases, then the corresponding reduction in prediction error. As shown in the first panel of the illustrated content, the system’s ability to self-evaluate its uncertainty has been successfully realized; however, there are still some imperfections with this model. The second panel shows some confusion patterns in the two sleep-related classes, indicating that there are some biological overlaps that can sometimes cause misclassification. Although the aggregated data show strength, the third panel still shows significant differences in individual error rates among patients.

**Figure 13:**
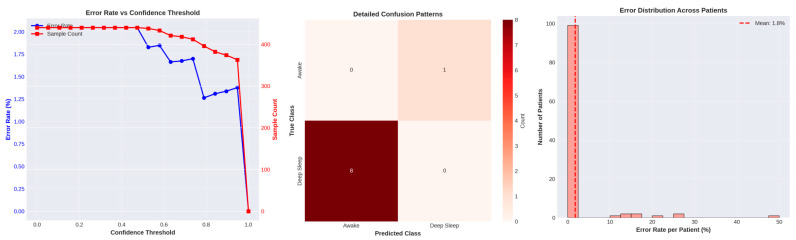
Comprehensive performance analysis of the model. (**Left**): Error rate vs. confidence threshold. (**Middle**): Detailed confusion patterns across awake and deep sleep classes. (**Right**): Error distribution across patients showing variability and outliers.

As shown in the left figure of Figure 13, the model’s performance remains relatively stable and consistently stays at approximately 2.0 percent across confidence thresholds up to 0.4. Only after reaching 0.5 do we observe some improvement in accuracy, though still declining slowly, and the inaccuracy is reduced to a relatively acceptable level of 1.2–1.5% around 0.7–0.8. However, the results at a threshold of 1.0 are, in fact, misleading; although the error rate technically drops to zero, this ideal state cannot be achieved due to the severe shortage of samples. From the strong pool of 420 cases to none at all is a tragicomedy that exposes a trade-off between achieving absolute certainty effectively and the model’s output.

The central confusion matrix shows that for the awake class, the model has one misclassified instance as deep sleep and no correct predictions, indicating a weak detection ability of this class. On the contrary, there is strong model confidence and accuracy in this state, as indicated by the deep sleep class eight correctly classified samples and none that are misclassified.

### 4.8. t-SNE Projection

To better understand how the GNN organizes patient-level representations, we produce a t-SNE projection of the learned node embeddings via visualization, as shown in two parts, to help reveal the structure of the embedding space and highlight areas related to classification errors. The left panel is the ground truth labels, while the right panel is the model’s predicted labels, including the mislabelled nodes.

**Figure 14:**
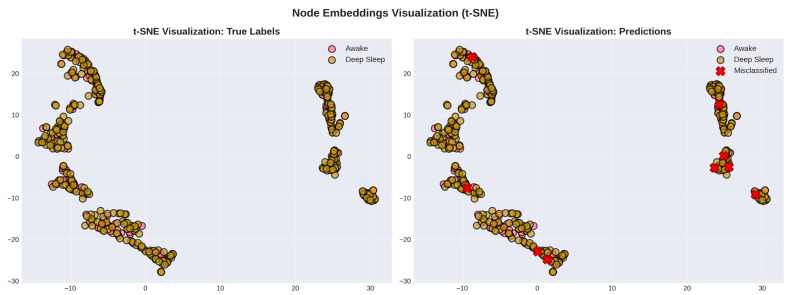
t-SNE visualization of node embeddings. (**Left**): True labels projected in 2D space. (**Right**): Predicted labels with misclassified nodes highlighted.

As shown in the left panel of Figure 14, the t-SNE projection still exhibits a clear clustering pattern for only two classes: awake and deep sleep. Most deep sleep nodes gather in several dense, tight clusters. On the other hand, these are more scattered and show an overlapping trend with nearby regions; therefore, although the deep sleep embeddings are relatively stable, the embeddings of the awake state are much more varied.

On the right, we have overlaid the model’s predictions in the same space and marked the misclassified nodes with red Xs. Most of these errors occur in the vicinity of the boundaries and transition areas between the awake and sleep states. We also list several misclassifications where awake points overlap with dense deep sleep clusters; this is because there are particular areas with high representation similarity that cause prediction confusion.

In general, the visualization shows that the GNN has learned an embedded manifold with structure but cannot completely separate borderline awake samples from deep sleep clusters, which is consistent with the misclassification pattern observed in the confusion matrix.

### 4.9. Comparison with Previous Studies

To contextualize the performance of the proposed framework, we compare it with representative prior works in the field of anesthesia depth monitoring. This allows us to assess the overall progress enabled by our approach, particularly in terms of accuracy and stability relative to traditional algorithms.

To ensure a fair and rigorous comparison, all methods listed in Table 4 were evaluated under a unified experimental strategy. Rather than directly adopting metrics from the original publications (which often addressed tasks of varying complexity, such as multi-state classification or different clinical endpoints), we re-evaluated or re-implemented these baseline algorithms on the exact same binary classification task (awake versus deep sleep) using the identical NTUH ECG dataset [25] and the same patient-level train/test splitting protocol. This strictly controlled setting guarantees that the observed performance differences reflect methodological and architectural advantages rather than discrepancies in task difficulty.

Table 4 summarizes the performance comparison of other methods and our proposed GNN model. Our model achieved the best result of 98.18%, which was more outstanding than all other studies. Jule Schübler et al. [44] reported 73.00%; Charles-Hervé Vacheron et al. [45] and Qihang Wang et al. [14] each accounted for 72.00% and 72.80%, respectively. These figures are much lower than those produced by our model. Andrew B. Barker et al. [46] achieved a higher accuracy of 75.20%, but it was still not as good as ours. Tai Nguyen-Ky et al. [47] have done the best work, achieving 90.54%, but our model was slightly better in the category of anesthesia depth.

## 5. Conclusions

Therefore, the GNN-based framework proposed in this paper for recognizing anesthesia levels based on ECG signals can achieve higher accuracy and robustness than CNN and other previous works. The design model makes full use of time-domain and non-linear features of ECG signals, as well as graph-based feature interactions; therefore, it can achieve good results. Although these good performances are encouraging, the following problems still exist. First, the dataset has only 110 patients, which may limit its generalizability to a broader group of people. Second, we only used ECG signals for analyses; if we combined other types of multi-modal signals, such as EEG and PPG, this could enhance classification performance. Third, although the presented strategy has achieved a significant result, further clinical trials are needed to verify its effectiveness under different surgeries and anesthesia in actual application for real-time implementation. Subsequent research will focus on expanding the framework to include multi-modal bio-signals, exploring adaptive GNN architectures, and researching federated-learning methods for improving privacy-protected model training across many institutions. Also, using explainable AI can make the decision-making process of the model more transparent to help build doctors’ confidence in using it. The proposed technology is capable of realizing more intelligent and non-intrusive anesthesia monitoring to improve surgical outcomes.

## Figures and Tables

**Table 1:** ECG features extracted for anesthesia depth classification with equation descriptions.

Feature Category	Feature	Equation	Description
Amplitude/Morphology	Mean Amplitude (μ)	$\mu =\frac{1}{N}\sum_{N}^{i=1}x_{i}$	Average signal value, represents overall ECG baseline [33].
	STD Amplitude (σ)	$\sigma =\sqrt{\frac{1}{N}\sum_{N}^{i=1}{\left(x_{i}-\mu \right)}^{2}}$	Measures signal variability around mean [34].
	Max Amplitude	$x_{\max}=\max(x_{i})$	Maximum peak amplitude in the segment [34].
	Min Amplitude	$x_{\min}=\min(x_{i})$	Minimum peak amplitude in the segment [34].
	Peak-to-Peak	$PTP=x_{\max}-x_{\min}$	Total amplitude range of the ECG waveform [35].
	Signal Energy	$E=\sum_{N}^{i=1}x_{i}^{2}$	Represents total power of the signal [36].
Power/Frequency	Signal Power	$P=\frac{1}{N}\sum_{N}^{i=1}x_{i}^{2}$	Average energy per sample, reflects signal strength [36].
	$\mathrm{Dominant}\;\mathrm{Frequency}\;(f_{d}$)	$f_{d}=\arg\max_{f}|X(f)|$	Frequency with maximum spectral amplitude, obtained from FFT [36].
Statistical/Complexity	Skewness	$Sk=\frac{\frac{1}{N}\sum_{i}{\left(x_{i}-\mu \right)}^{3}}{\sigma^{3}}$	Measures asymmetry of signal distribution [37].
	Kurtosis	$K=\frac{\frac{1}{N}\sum_{i}{\left(x_{i}-\mu \right)}^{4}}{\sigma^{4}}$	Measures peaks or flatness of signal distribution [37].
	Sample Entropy	$\mathrm{SampEn}=-\ln\frac{A}{B}$	Quantifies signal complexity and irregularity [38].
	Zero-Crossing Rate	$ZCR=\frac{1}{N-1}\sum_{N-1}^{i=1}{⊬}_{\left\{x_{i}x_{i+1}<0\right\}}$	Rate of sign changes, indicates frequency content [34].
	Spectral Entropy	$H=-\sum_{f}P(f)\log P(f)$	Measures randomness in frequency domain [34].
HRV/Autonomic	Mean HR	$\mathrm{HR}=\frac{60}{mean\mathrm{RR}interval}$	Average heart rate from RR intervals [39].
	SDNN	$\mathrm{SDNN}=\sqrt{\frac{1}{N-1}\sum {\left(RR_{i}-\bar{RR}\right)}^{2}}$	Overall HRV, standard deviation of RR intervals [39].
	RMSSD	$\mathrm{RMSSD}=\sqrt{\frac{1}{N-1}\sum {\left(RR_{i+1}-RR_{i}\right)}^{2}}$	Short-term HRV, reflects parasympathetic activity [40].
	Mean RR Interval	$\bar{RR}=\frac{1}{N}\sum RR_{i}$	Average time interval between consecutive R-peaks [41].
	LF Power	$P_{LF}=\sum_{f\in LF}|X(f){|}^{2}$	Power in low-frequency band, reflects sympathetic activity [42].
	HF Power	$P_{HF}=\sum_{f\in HF}|X(f){|}^{2}$	Power in high-frequency band, reflects parasympathetic activity [42].
	LF/HF Ratio	$LF/HF=\frac{P_{LF}}{P_{HF}}$	Balance between sympathetic and parasympathetic activity [42].

**Table 2:** Hyperparameter experiment results.

Category	Parameter	Accuracy (%)	Training Time per Step (ms)
Convolutional Layers	3 layers	83.91	128 ms
	2 layers	84.22	132 ms
	4 layers	83.73	140 ms
Hidden Units	64, 64	86.41	128 ms
	32, 32	83.92	8 s
Combination Type	Concat	84.48	128 ms
	ConvLSTM1D	86.99	128 ms
Activation Function	ELU	87.55	128 ms
	ReLU	81.44	128 ms
	Tanh	82.11	128 ms
	Softmax	84.44	128 ms
Optimizer	Adam	84.22	128 ms
	Nadam	85.14	128 ms
	Adamax	80.91	128 ms
	SGD	88.01	128 ms
Learning Rate	0.01	89.66	128 ms
	0.001	83.47	20 ms
	0.0001	85.77	20 ms
	0.0007	65.78	6 s
Dropout	0.3	91.01	128 ms
	0.4	84.10	72 ms
	0.5	78.59	20 ms
Batch Size	32	82.87	74 ms
	64	91.46	7 s
	128	88.11	128 ms

**Table 3:** Performance metrics comparison between 1D CNN, GNN, and knowledge GCN variants.

Performance Metrics	1D CNN	Knowledge GCN	Enhanced Knowledge GCN	Deep Knowledge GCN (Best)
Test Accuracy	79.41	97.95	71.36	98.18
Sensitivity	66.31	97.20	70.10	98.40
Precision	78.37	98.10	72.30	98.50
Specificity	79.41	98.40	72.10	98.60
NPV	94.87	99.10	85.20	99.30
FPR	19.22	1.60	27.90	1.40
FDR	21.43	1.90	27.70	1.50
FNR	17.29	2.80	29.90	1.60
F1-Score	79.21	97.60	71.10	98.45
MCC	72.31	97.10	69.40	98.00

**Table 4:** Performance Comparison with Existing Methods.

Method	Performance (%)
Ours	98.18
Jule Schübler et al.	73.00
Charles-Hervé Vacheron et al.	72.00
Qihang Wang et al.	72.80
Andrew B. Barker et al.	75.20
Tai Nguyen-Ky et al.	90.54

## Data Availability

The dataset utilized in this study comprises intraoperative physiological data collected from 110 patients undergoing general anesthesia at the National Taiwan University Hospital (NTUH).
